# Anoctamin 9 determines Ca^2+^ signals during activation of T-lymphocytes

**DOI:** 10.3389/fimmu.2025.1562871

**Published:** 2025-03-26

**Authors:** Rainer Schreiber, Jiraporn Ousingsawat, Karl Kunzelmann

**Affiliations:** Physiological Institute, University of Regensburg, Regensburg, Germany

**Keywords:** lymphocytes, T-cells, T-cell receptor, TCR, immunological synapse, anoctamin 9, ANO9, TMEM16J

## Abstract

**Background:**

Activation of T-cells is initiated by an increase in intracellular Ca^2+^, which underlies positive and negative regulation. Because the phospholipid scramblase and ion channel ANO9 (TMEM16J) was shown previously to regulated Ca^2+^ signals in renal epithelial cells, we asked whether ANO9 demonstrates a similar regulation in T-cells.

**Methods:**

We used measurements of the intracellular Ca^2+^ concentration to examine the effects of ANO9 on intracellular Ca^2+^ signaling and demonstrated expression of ANO9 and its effects on cellular and molecular parameters.

**Results:**

ANO9 was found to be expressed in human lymphocytes, including the Jurkat T-lymphocyte cell line and mouse lymphocytes. ANO9 has been shown to affect intracellular Ca^2+^ signals in renal epithelial cells. Here we demonstrate the essential role of ANO9 during initiation of intracellular Ca^2+^ signals in Jurkat T-cells and isolated mouse lymphocytes. ANO9 is essential for the initial rise in intracellular Ca^2+^ due to influx of extracellular Ca^2+^ through store-operated ORAI1 Ca^2+^ entry channels. ANO9 is indispensable for T-cell function, independent on whether cells are activated by stimulation of the T-cell receptor with CD3-antibody or by PMA/phytohemagglutinin.

**Conclusions:**

Upon activation of T-cells and formation of the immunological synapse, ANO9 recruits the Ca^2+^-ATPase (PMCA) to the plasma membrane, which is supported by the scaffolding protein discs large 1 (DLG1). PMCAs maintain low Ca^2+^ levels near ORAI1 channels thereby suppressing Ca^2+^-inhibition of ORAI1 and thus retaining store-operated Ca^2+^ entry (SOCE). It is suggested that ANO9 has a role in interorganelle communication and regulation of cellular protein trafficking, which probably requires its phospholipid scramblase function.

## Introduction

The first event in the activation of T cells by stimulation of the T cell receptor (TCR) is an increase in intracellular Ca^2+^, which is essentially caused by Ca^2+^ influx through store-operated ORAI channels (1). Activation of the T-cell receptor and formation of phosphatidylinositol 4,5-bisphosphate (PIP_2_) releases Ca^2+^ from the endoplasmic reticulum (ER) Ca^2+^ store, which is sensed by stromal interaction molecule 1 (STIM1) leading to activation of ORAI1, the pore-forming subunit of the Ca^2+^ release-activated Ca^2+^ influx channel (CRAC) (2–5). Thus, missense mutations in ORAI1 or Stim1 lead to severe immune deficiency (6, 7). While Ca^2+^ influx occurs mainly through ORAI1 and is modulated by numerous additional factors, additional Ca^2+^ entry may occur through transient receptor potential (TRP) channels, voltage gated Ca^2+^ channels and P2X receptors (1).

ANO9 (anoctamin 9, TMEM16J) is a phospholipid (PL) scramblase and a cation channel (8–10), which was shown to amplify olfactory transduction in mammalian olfactory sensory neurons (11). A pathogenic role of ANO9 for chronic kidney disease (CKD) has been suggested in a genome-wide meta-analysis for creatinine-based estimated glomerular filtration rate, and identified a variant for ANO9 (12). The corresponding mutant ANO9-T604A was shown to cause a constitutive release of interleukin 6 and 8 by renal epithelial cells (10).

ANO9 is an activator of a disintegrin and metalloprotease (ADAM) 17 that may contribute to CKD through its PL-scrambling activity (13, 14). Overexpression of ANO9 in HEK293 cells activated plasma membrane (PM) Ca^2+^ pumps (PMCA), enhanced basal Ca^2+^ influx through ORAI1 and attenuated ER Ca^2+^ store release triggered by stimulation of G-protein coupled receptors (GPCRs) (10, 15). Renal expression of ANO9 in healthy kidneys is relatively low, and we therefore ask whether the low expression of ANO9 detected by RNAseq analysis of immune cells may affect intracellular Ca^2+^ signaling and thereby contribute to CKD (Human Protein Atlas proteinatlas.org). While CD4+ and CD8+ T-lymphocytes clearly express ANO9, it is these T-cell populations that are of major importance for acute kidney injury and CKD (16, 17). Fundamental aspects of T-cell signaling have been identified in Jurkat T-cells, which were also used in the present study, along with freshly isolated mouse lymphocytes (18). We demonstrate a fundamental role of ANO9 for initiation of the initial Ca^2+^ response in Jurkat CD4+ lymphocytes. The data indicate ANO9 as a regulator of PMCA expression in the plasma membrane, which is a crucial determinant of ORAI1-mediated Ca^2+^ influx during activation of T-cells.

## Results

### ANO9 expressed in Jurkat T-cells induces a whole cell current and PL scramblase activity

**Figure 1:**
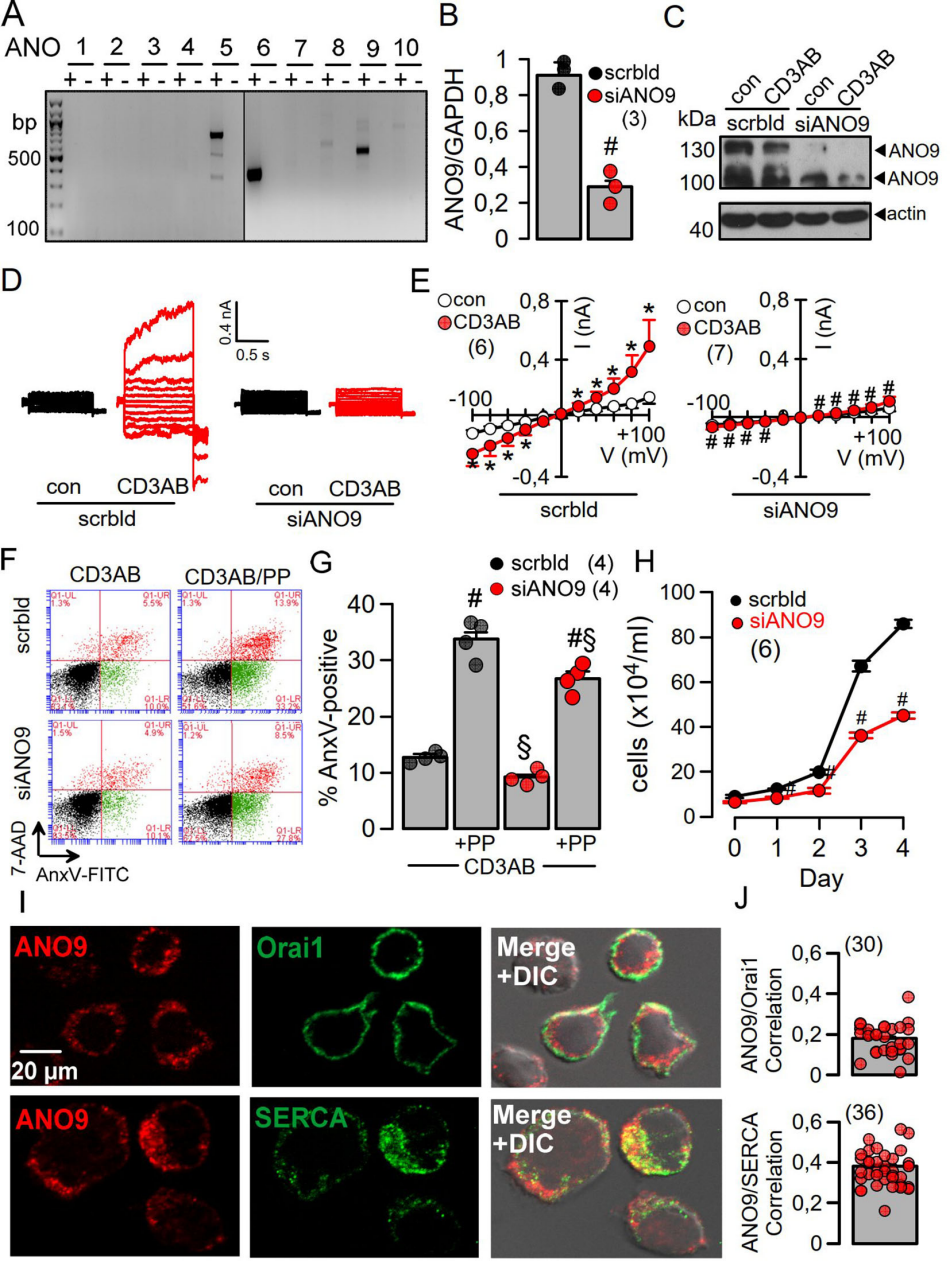
ANO9 ion currents and phospholipid scrambling is activated during activation of Jurkat T-lymphocytes. **(A)** RT-PCR analysis showing expression of ANO5, 6 and 9 in Jurkat T-cells. **(B)** Semiquantitative RT-PCR analysis of ANO9 expression indicating knockdown by siRNA-ANO9. **(C)** Western blot of ANO9 (130/100 kDa) before and after activation by CD3AB (2.5 µg/ml) and knockdown by siRNA-ANO9. (scrbld; treatment with scrambled RNA). **(D)** Currents overlays indicating activation of whole cell currents in Jurkat T-cells by CD3AB (2.5 µg/ml), which is absent in cells treated with siRNA for ANO9 (siANO9). **(E)** Corresponding current/voltage relationships. **(F)** FACS analysis of cells activated with CD3-AB or CD3-AB and phorbol-12-myristate-13-acetate and phytohemagglutinin (PP; 5 ng/mL/10 µg/mL) (CD3/PP) indicates partial inhibition of annexin V (AnxV) exposure by siANO9. 4.8 ± 0.9% (scrbld) and 3.7 ± 0.6% (siANO9) of the nonstimulated control cells were AnxV positive. **(G)** Summary of percentage annexin V (AnxV) positive cells. **(H)** Effect of siRNA-ANO9 on Jurkat T-cell proliferation. **(I)** Co-staining of ANO9 together with ORAI1 or SERCA in Jurkat T-cells. **(J)** Colocalization estimated by calculated Pearson’s correlation coefficient (c.f. Methods). Mean ± SEM (number of experiments). *significant activation by CD3AB (p < 0.05; paired t-test). ^#^significant effect of siANO9 and PP, respectively (p < 0.05; unpaired t-test). ^§^significant inhibition by siANO9 (p < 0.05; unpaired t-test).

Using RT-PCR we identified expression of the anoctamins ANO5, 6, and 9 in Jurkat cells (**Figures 1A, B**) Expression of ANO9 could be downregulated using siRNA, as shown by semiquantitative RT-PCR, Western blotting, and immunostaining (**Figures 1B, C**, **Supplementary Figure 1**). A whole cell (wc) current was activated by stimulation of the T-cell receptor with a CD3-antibody (CD3AB), consisting of ORAI1 Ca^2+^ currents along with K^+^ and Cl^-^ currents, as shown previously (19, 20). Activation of wc currents was completely abolished after siRNA-knockdown of ANO9 (siANO9), suggesting a central role of ANO9 during activation of T-cells (**Figures 1D, E**). ANO9 is a phospholipid scramblase causing exposure of phosphatidylserine (PS) in the outer leaflet of the plasma membrane. PS exposure can be detected in flow cytometry by binding of fluorescent annexin V (AnxV), while membrane permeable 7-AAD detected apoptotic cells. Stimulation by CD3AB induced minimal PL-scrambling, which, however, was further augmented by additional stimulation with phorbol-12-myristate-13-acetate (PMA) and phytohemagglutinin (PHA) (PP). PL-scrambling was only partially inhibited by knockdown of ANO9, supporting a major role of the PL-scramblase ANO6 (21), while cell proliferation was strongly suppressed by siANO9 (**Figures 1F–H**). ANO9 was co-stained together with ORAI1 or SERCA in Jurkat T-cells, and colocalization was estimated by Pearson’s correlation coefficient (**Figures 1I, J**).

### ANO9 is essential for the initial increase in intracellular Ca^2+^ during activation of Jurkat T-cells

**Figure 2:**
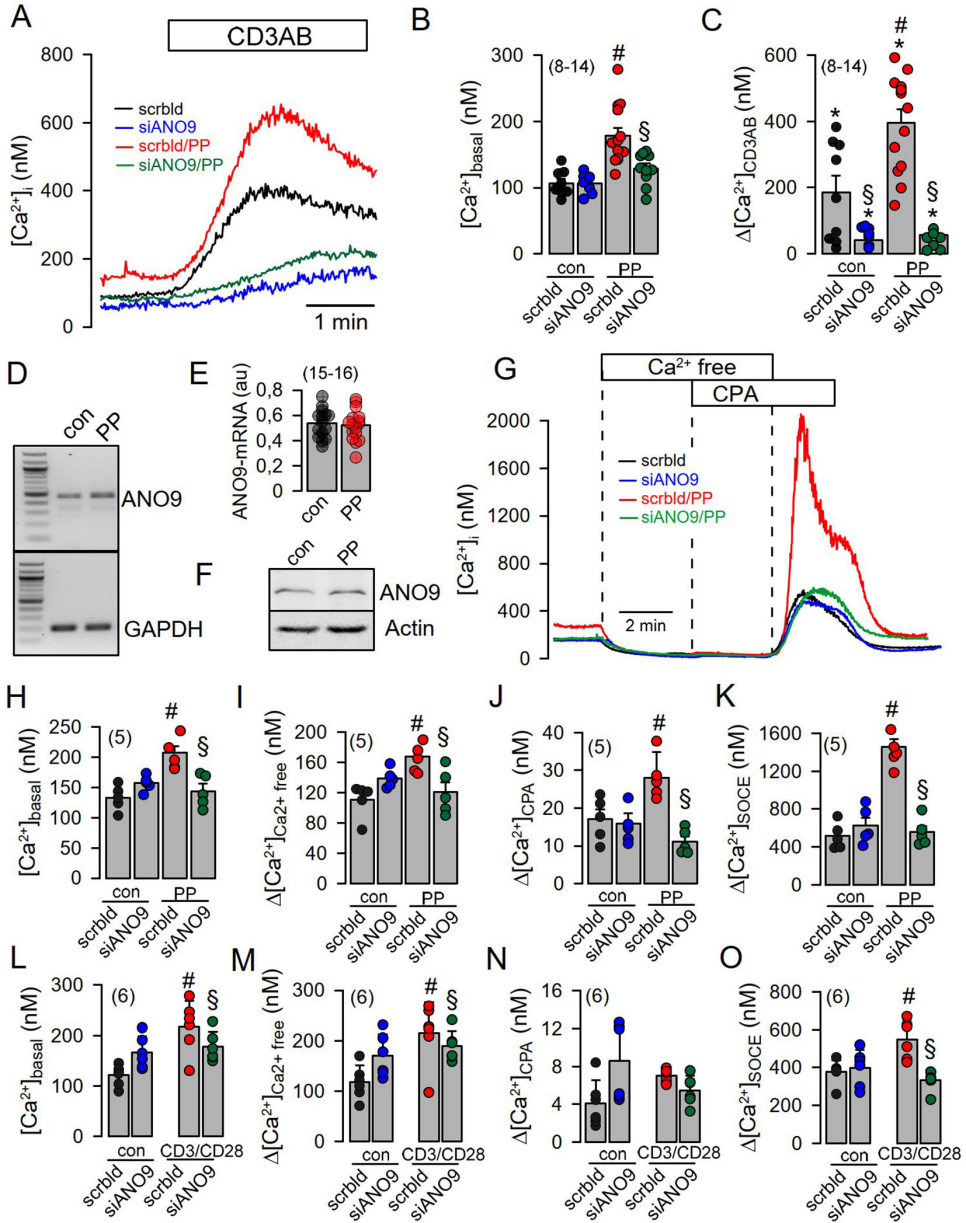
Increase in [Ca^2+^]_i_ during activation of Jurkat T-cells requires ANO9. **(A)** Increase of intracellular Ca^2+^ ([Ca^2+^]_i_ *)* by acute (CD3AB) and chronic (PP; 5 ng/mL/10 µg/mL for 24 hrs) activation in the absence or presence of siRNA-ANO9. **(B)** Summary of siRNA-ANO9 knockdown on basal [Ca^2+^]_i_ in the absence or presence of PP. **(C)** Summary of siRNA-ANO9 knockdown on [Ca^2+^]_i_ increase induced by CD3AB and PP. **(D, E)** Analysis by semiquantitative RT-PCR of the effect of PP on expression of ANO9. **(F)** Western blot of ANO9 in the absence or presence of PP-stimulation. **(G)** Effects of extracellular Ca^2+^ removal and cyclopiazonic acid (CPA; 10 µM) on [Ca^2+^]_i_ under basal conditions and after PP-activation. Summaries of siRNA-ANO9 knockdown on basal [Ca^2+^]_i_ **(H)**, Ca^2+^ removal **(I)**, CPA **(J)**, and SOCE **(K)** in the absence or presence of PP. **(L-O)** Summaries of siRNA-ANO9 knockdown on basal [Ca^2+^]_i_, Ca^2+^ removal, CPA, and SOCE in the absence or presence of CD3AB. Mean ± SEM (Number of cover slips. On each cover slip about 10-15 cells were measured). ^#^significant increase by PP (p < 0.05; unpaired t-test). *significant increase by CD3AB (p < 0.05; paired t-test). ^§^significant effect of siANO9 (p < 0.05; unpaired t-test).

**Figure 3:**
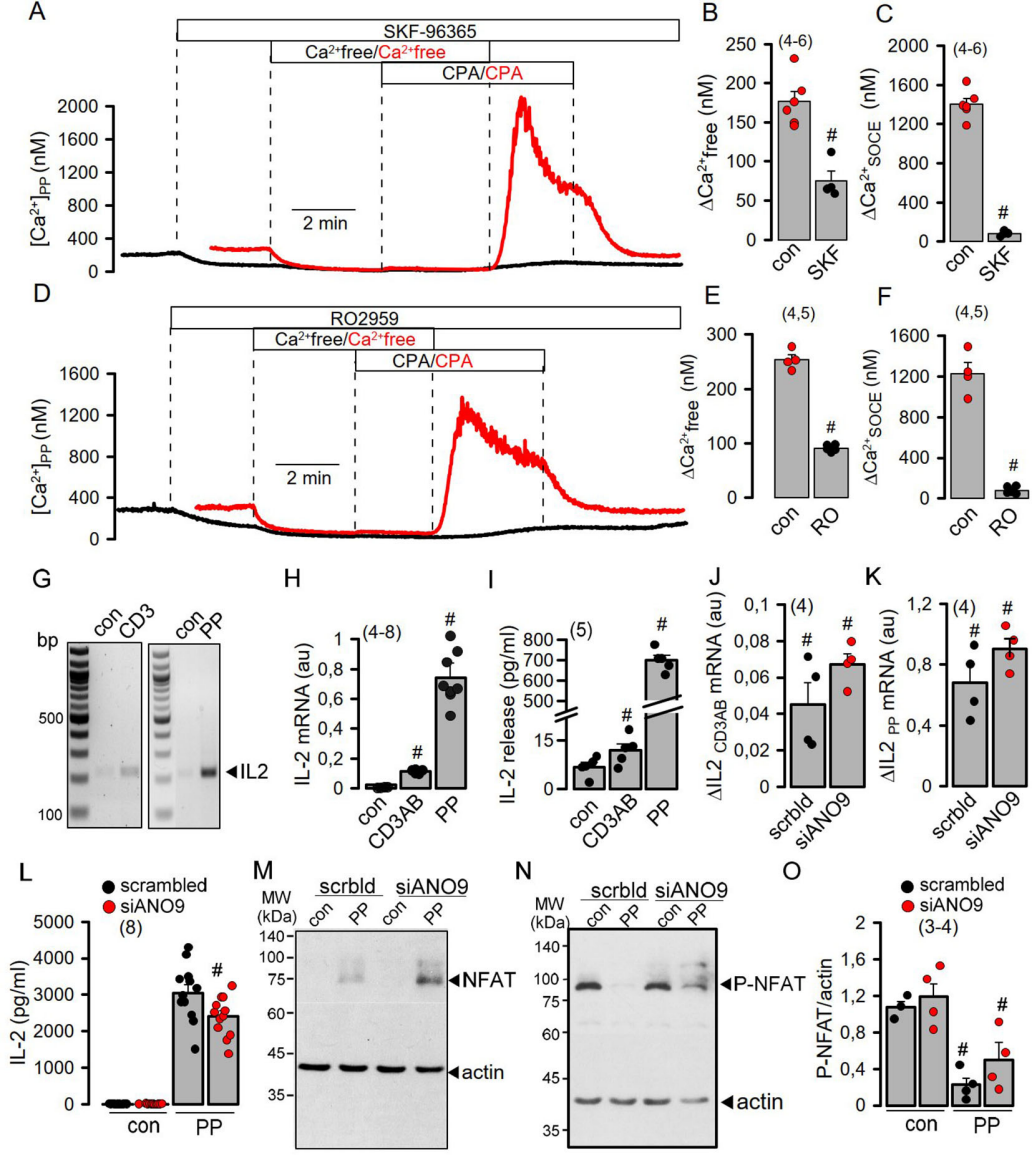
SOCE induced by activation of Jurkat T-cells is disconnected from IL-2 release by ANO9. **(A-F)** Inhibition of basal Ca^2+^ influx (ΔCa^2+^-free) and SOCE (ΔCa^2+^-SOCE) in PP- (5 ng/mL/10 µg/mL for 24 hrs) activated Jurkat T-cells by the Orai-inhibitors SKF-96365 (10 µM) and RO2959 (5 µM). (Number of cover slips. On each cover slip about 10-15 cells were measured). **(G, H)** Semiquantitative RT-PCR analysis of the induction of IL-2 expression by CD3AB (2.5 µg/ml) and PP. **(I)** Induction of IL-2 release by CD3AB and PP. **(J, K)** Semiquantitative RT-PCR analysis indicates no effect of siRNA-ANO9 on expression of IL-2 induced by CD3AB or PP. **(L, M)** Western blots for NFAT and P-NFAT before and after PP-stimulation in the absence or presence of siRNA-ANO9. **(N)** Summary of the effects of siRNA-ANO9 on PP-induced dephosphorylation of NFAT. **(O)** Effect of siRNA-ANO9 on IL-2 secretion. Mean ± SEM (number of experiments). ^#^significant effects of SKF-96365, RO2959, and stimulation by CD3AB and PP, respectively (p < 0.05; unpaired t-test).

Co-staining of ANO9 with ORAI1 and the ER Ca^2+^-ATPase (SERCA) suggested colocalization of ANO9 with proteins that regulate intracellular Ca^2+^-signals (**Figure 1I**). Using the Ca^2+^ sensor Fura2, we detected a transient Ca^2+^ increase induced by stimulation of the T-cell receptor with CD3AB. CD3AB-induced rise in intracellular Ca^2+^ ([Ca^2+^]i) was further enhanced in the presence of PP, which also augmented basal [Ca^2+^]i (**Figures 2A–C**). Basal and CD3AB-enhanced [Ca^2+^]i were completely blocked by siANO9 (**Figures 2A–C**). Importantly, activation by PP did not change expression of ANO9 in Jurkat T-cells as demonstrated by RT-PCR and Western blotting (**Figures 2D–F**). We further determined the role of ANO9 for Ca^2+^ signaling by removing extracellular Ca^2+^ and emptying the ER Ca^2+^ store using cyclopiazonic acid (CPA), a reversible SERCA inhibitor. Because direct stimulation of TCR can be bypassed by PP, we continued by activating Jurkat T-cells with PP only (18, 22). PP augmented basal Ca^2+^ influx and massively upregulated Ca^2+^ influx after emptying the store by CPA and re-addition of extracellular Ca^2+^ (**Figure 2G**). PP-induced upregulation of Ca^2+^ influx was entirely inhibited by knockout of ANO9 expression (**Figures 2G, H–K**). Moreover, similar results were obtained when Jurkat T-cells were activated by stimulation of the T-cell receptor with CD3 antibody (**Figures 2L–O**). The data indicate that ANO9 is indispensable for T-cell function, regardless of whether cells are activated by stimulation of the T-cell receptor with CD3-antibody or by PMA/phytohemagglutinin (PP). Remarkably, there was only a negligible increase in cytosolic Ca2+ when the store was emptied by CPA, indicating very efficient clearance of cytosolic Ca^2+^ after being released from the store, likely due to the plasma membrane Ca^2+^-ATPase (PMCA) (23, 24). We did not find evidence for a change in expression of ORAI1, inositol trisphosphate receptors 1-3 (IP_3_R1-3) or ryanodine receptor 2 (RYR2) during PP-activation or knockdown of ANO9, apart from of a small increase in expression of RYR3 (**Supplementary Figures 2A–F**). As another possibility, ANO9 may directly regulate ORAI1 activity or it may control PM-expression of ORAI1, which has been examined below. In any case, the large increase in store operated Ca^2+^ influx upon stimulation of Jurkat T-cells was entirely due to ORAI1, as the ORAI-inhibitors SKF-96365 and RO2959 completely inhibited SOCE (**Figures 3A–F**). Moreover, the anoctamin inhibitor niclosamide (25) strongly attenuated Ca^2+^ increase, SOCE and IL-2 release induced by CD3AB or PP, while other anoctamin inhibitors like Ani9, niflumic acid (NFA), or tannic acid (TA) showed no effects (**Supplementary Figure 3**). This is probably explained by the fact that niclosamide has a pronounced inhibitory effect on intracellular Ca^2+^, apart from directly inhibiting anoctamin proteins. Taken together, knockdown of ANO9 completely abolished store operated Ca^2+^ entry (SOCE) through ORAI1 channels.

### Although ANO9 is essential for Ca^2+^ increase, its contribution to the subsequent signaling cascade is limited

**Figure 4:**
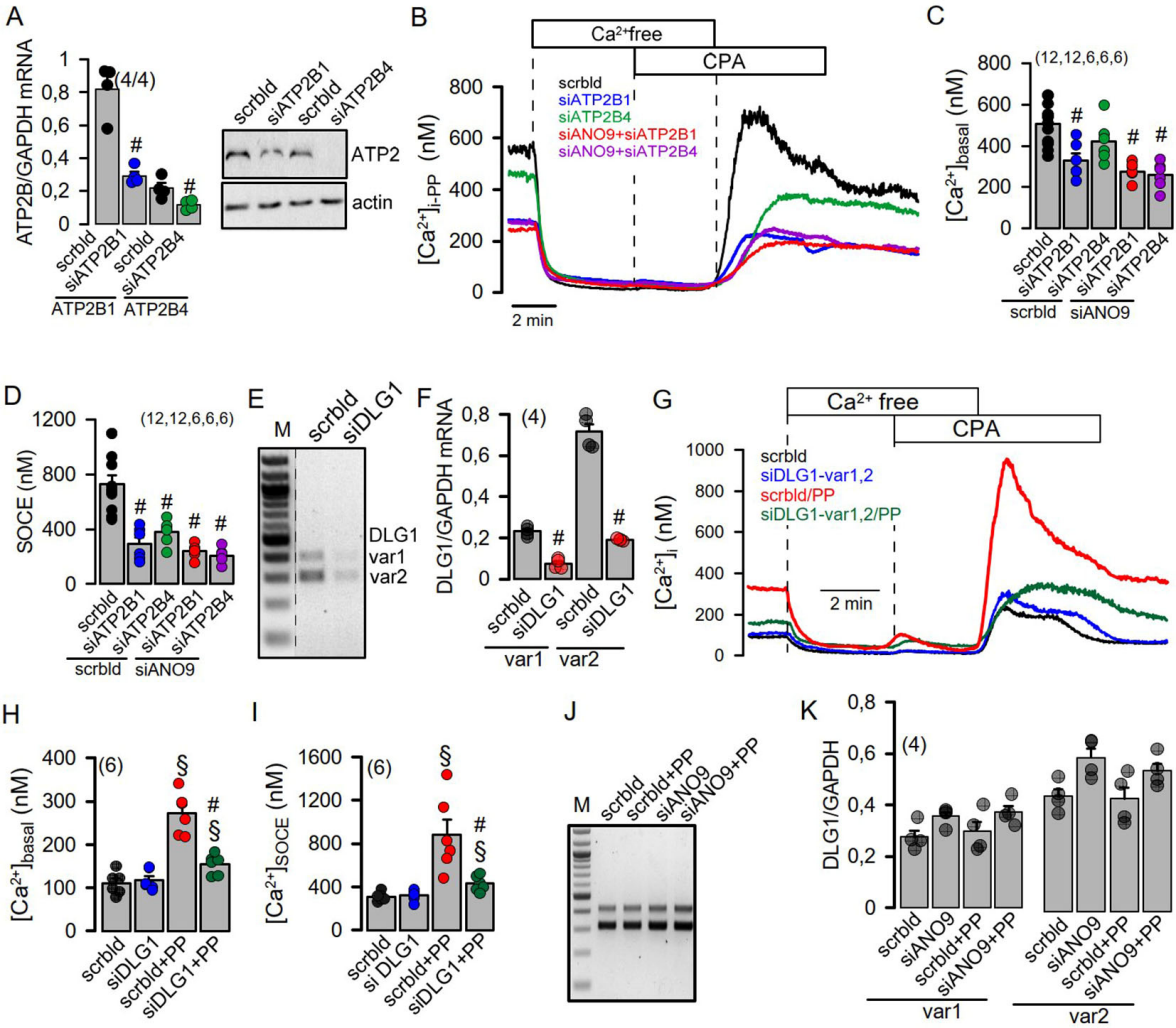
PMCA (ATP2B) and the scaffold DLG1 are required to maintain SOCE. **(A)** Summary of the semiquantitative RT-PCR analysis of the effect of siRNA-knockdown on the expression of the PMCAs ATP2B1 and ATP2B4 in Jurkat T-cells (left) and confirmation of the knockdown of ATP2B1 and ATP2B4 protein by Western blotting (right). **(B-D)** Effect of the siRNA-knockdown of ATP2B1, ATP2B4 and ANO9 on basal Ca^2+^ influx and SOCE in PP activated cells. **(E, F)** Summary of the semiquantitative RT-PCR analysis of siRNA-knockdown of DLG1 variants in Jurkat T-cells. **(G-I)** Effect of siRNA-knockdown of DLG variants on basal Ca^2+^ influx and SOCE. **(J, K)** Lack of effects of PP-stimulation or siRNA-ANO9 on expression of DLG1 variants. Mean ± SEM (Number of cover slips. On each cover slip about 10-15 cells were measured). ^#^significant inhibition by si-ATP2B and si-DLG1, respectively (p < 0.05; unpaired t-test). ^§^significant effect of PP (p < 0.05; unpaired t-test).

Activation of Jurkat T-cells by PP strongly enhanced expression and release of interleukin 2 (IL-2) compared to only small increases induced by CD3AB (**Figures 3G–I**). While transcription of IL-2 was not affected by knockdown of ANO9, dephosphorylation of the nuclear factor of activated T-cells (NFAT) and release of IL-2 were only slightly inhibited, despite complete inhibition SOCE (**Figures 3J–O**). Plasma membrane Ca^2+^-ATPases (PMCAs) are known positive and negative modulators of Ca^2+^ signaling in lymphocytes. They are critical for removal of cytosolic Ca^2+^ to maintain SOCE (23, 26), but also uncouple expression of IL-2 from early TCR Signaling (27). As anoctamins were shown to control intracellular Ca^2+^ signaling (28, 29), we examined whether ANO9 may affect the function of PMCAs in activated Jurkat cells. The two major PMCAs expressed in these cells, ATP2B1 and ATP2B4, were knocked down by siRNA, which strongly inhibited basal [Ca^2+^]i and SOCE in PP-activated cells (**Figures 4A–D**). Additional knockdown of ANO9 did not further attenuate SOCE, which suggests that ANO9 acts through PMCA to maintain Ca^2+^ influx.

### ANO9 may engage DLG1 to translocate PMCA to the plasma membrane

The scaffolding protein discs large 1 (DLG1) recruits the PMCA to the plasma membrane (30, 31). As ANO9 also interacts with DLG1 (32), we hypothesized that ANO9 is essential to translocate the PMCA to the plasma membrane and the immediate vicinity of the immunological synapse (33). We knocked-down the two DLG1 variants expressed in Jurkat cells, which essentially abolished basal Ca^2+^ influx and SOCE (**Figures 4E–I**). As knockdown of ANO9 did not inhibit transcription of DLG1 we proposed a functional role of ANO9/DLG1 for insertion of PMCA into the plasma membrane (**Figures 4J, K**). We immunolabeled ORAI1 and PMCA in Jurkat cells before and after activation by PP and quantified the fluorescence signal. Staining of ORAI1 was unaffected by stimulation with PP or siRNA-knockdown of ANO9. In contrast, fluorescence labelling of PMCA in the plasma membrane was increased after PP-activation, and this increase was attenuated in cells knocked-down for ANO9 (**Supplementary Figure 4**). Likewise, knockdown of DLG1 had no effect on ORAI1 staining, but reduced plasma membrane staining of PMCA in PP-activated cells (**Supplementary Figure 5**). These data suggest a central role of ANO9 and DLG1 for maintaining Ca^2+^ entry in activated T-cells, through targeting of PMCA to the plasma membrane.

### ANO6 but not ANO9 regulates expression of PD-1 or PD-L1

**Figure 5:**
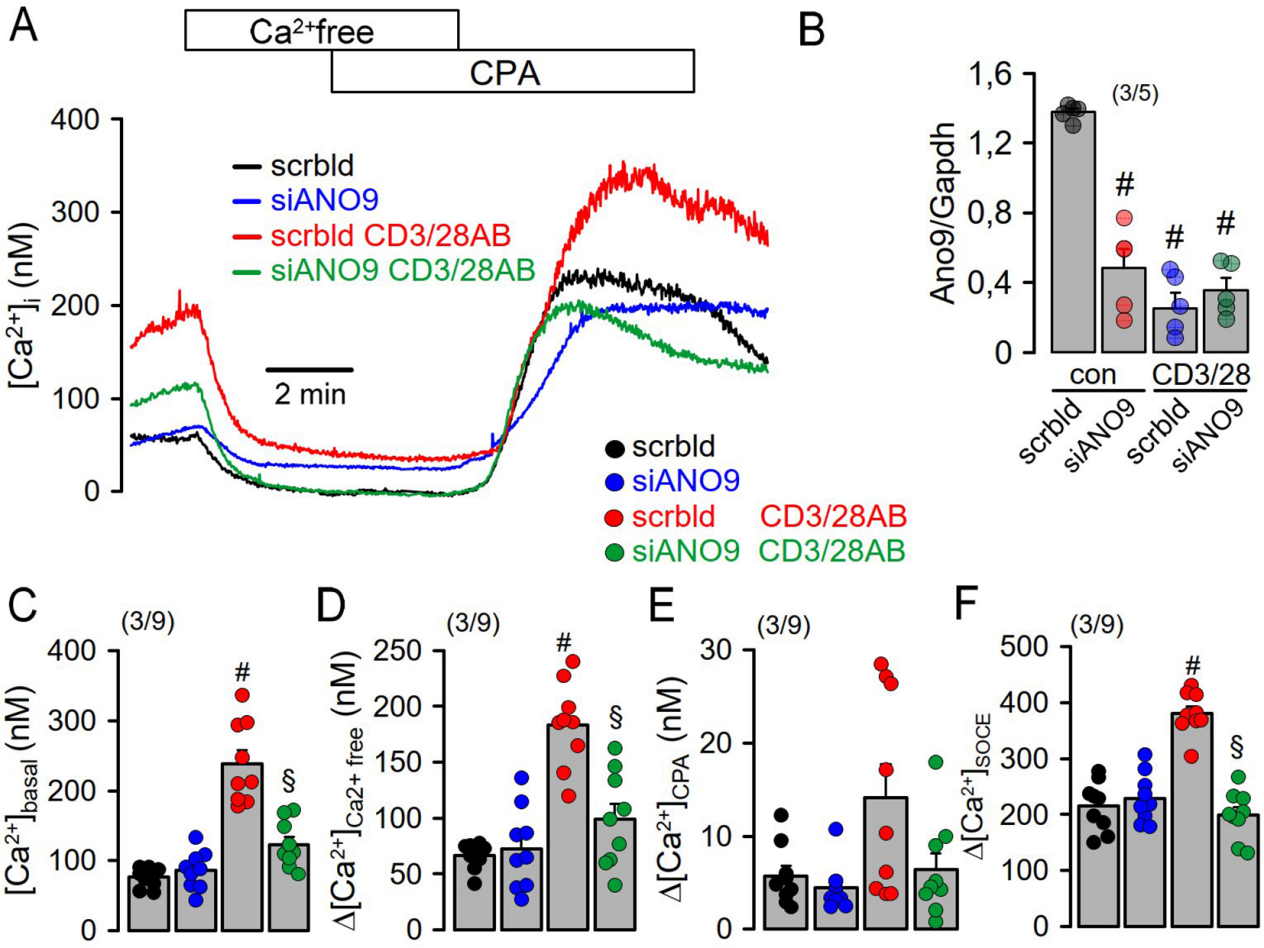
Increase in [Ca^2+^]_i_ during activation of freshly isolated mouse lymphocytes requires ANO9. **(A)** Effects of extracellular Ca^2+^ removal and cyclopiazonic acid (CPA; 10 µM) on [Ca^2+^]_i_ of mouse lymphocytes under control conditions (con) and after activation by CD3 and CD28 antibodies (CD3/28AB). **(B)** siRNA-knockdown of ANO9 expressed in mouse lymphocytes in the absence or presence of CD3/28AB. **(C)** Summary of the effects of CD3/28AB and siRNA-ANO9 on basal [Ca^2+^]i **(D, E)**. Summary of the effects of extracellular Ca^2+^ removal and cyclopiazonic acid (CPA; 10 µM) on [Ca^2+^]i. **(F)** Effects of re-addition of extracellular Ca^2+^ on SOCE under control conditions (con) and after CD3/28AB-activation. Mean ± SEM (number of animals/cells measured). ^#^significant increase by CD3/28AB (p < 0.05; ANOVA). ^§^significant effect of siANO9 (p < 0.05; ANOVA).

In T-cells ANO6 is the dominating phospholipid scramblase (34) (**Figure 1**). ANO6 promotes exocytosis of programmed cell death 1 (PD-1) which terminates immune responses, while ANO9 might control expression of PD-1 receptor ligands (PD-L1,2) (21, 34, 35). We found that release of IL-2 was enhanced by knockdown of ANO6, but was suppressed by knockdown of ANO9, supporting previous observations (34) (**Supplementary Figure 6A–C**). PD-1 and PD-L1 are expressed in Jurkat cells and PP-activation strongly upregulated expression of PD-1 and PD-L1, which, however, was not affected by knockdown of ANO9 (**Supplementary Figure 6D–H**). Thus, different anoctamin scramblases may have distinct functions in T-cells. In summary, the present data suggest an essential role of ANO9 in maintaining store-operated Ca^2+^ entry during the initial step of Jurkat T-cell activation, a role that was also confirmed in freshly isolated mouse lymphocytes (**Figure 5**). Although we detected an only weak PL scramblase activity of ANO9 in flow cytometry (**Figures 1F, G**), intracellular scrambling by cytosolic ANO9 may support insertion of the PMCA into the plasma membrane.

## Discussion

The present data indicate a fundamental role ANO9 in controlling the initial Ca^2+^ increase upon activation of T-cells and formation of the immunological synapse (36). The absence of ANO9 abolished the initial rise in cytosolic Ca^2+^ upon activation of human Jurkat T-cells and mouse lymphocytes, and essentially abolished store-operated Ca^2+^ entry (SOCE). While SOCE was entirely dependent on ANO9, it had a limited impact on IL-2 release, suggesting additional mechanisms that regulate peripheral tolerance due to induction of anergy-associated genes (37). Thus, PMCA4 was shown to uncouple IL-2 expression from early TCR signaling, which is discussed below (27). However, since ANO9 shows such a pronounced effect on the activation of SOCE, i.e. ORAI1 activity, ANO9 itself could be a target of regulated tolerance and anergy. Indeed, we observed that TCR activation in Jurkat cells by CD3AB inhibited the expression of ANO9 (**Figure 1C**), and a similar inhibition of ANO9 expression was observed during CD3AB/CD28AB stimulation in isolated mouse T cells (**Figure 5B**). This observation underscores the importance of ANO9-dependent regulation of Ca^2+^ signaling in T cells.

PMCAs are key players in fine-tuning the magnitude and duration of [Ca^2+^]i signals. Upon activation of T-cells, DLG1 recruits PMCA to the plasma membrane (30, 31), while ANO9 interaction with DLG1 supports translocation of PMCA to the immediate vicinity of the immunological synapse (32, 33) (**Supplementary Figure 7**). PMCA enables sustained SOCE by maintaining a low [Ca^2+^]_i_ in a functional compartment containing ORAI1, PMCA4, Stim1 and partner of Stim1 (Post) (23, 24, 26, 38). In contrast to SOCE, store release Ca^2+^ is extremely well buffered due to immediate uptake into mitochondria via mitochondrial Ca^2+^ uniporter (MCU) located in mitochondria-associated membranes (MAMs) (1).

DLG1 is engaged in the traffic of ion channels, pumps, and signaling proteins to specialized zones within the plasma membrane (39). DLG1 interacts with pleckstrin homology and FYVE domain containing 2 (PLEKHF2), a protein that is required for early endosome fusion upstream of RAB5 (32, 40). This suggests a function of ANO9 similar to that of its close paralog ANO10 (TMEM16K). ANO10 was shown to form contacts with Rab7-positive endosomes and facilitates endosomal sorting through interorganelle communication (41). In experiments with split-GFP, Petkovic et al. found evidence that the lipid-scrambling activity of TMEM16K is required for endosomal sorting (41). We therefore hypothesize that phospholipid scrambling by ANO9 in intracellular vesicles may support targeting of PMCA to the immunological synapse.

ANO9 supported proliferation of Jurkat T-lymphocytes, corresponding to its well-known role in cancer development (42). In T-cells pro-proliferative effects of ANO9 may be partly due to its negative effects on programmed cell death 1 (PD-1) signaling (35, 43). PD-1 is a cell surface receptor that downregulates the immune response in T-cells to avoid immune exhaustion (44). Activation of Jurkat T-cells stimulates the PL-scramblase ANO6 which causes shedding of PD-1 containing ectosomes (21). Correspondingly, mice lacking expression of ANO6 suffer from immune exhaustion, which results in a failure to clear viral infections (34). Hu et al. localized ANO6 in endosomes near the immunological synapse of T-cells and proposed that ANO6 controls PD-1 surface expression. PL-scrambling negatively regulates NK cell activation (45), while in T-cells ANO6 recruits bystander TCR-CD3 and numerous other proteins to the plasma membrane to regulate TCR-dependent signaling (46, 47). Taken together, ANO9 (and ANO6) have stimulatory and inhibitory functions in lymphocytes. Modulation of T-cell function by ANO9 could contribute to its role in a variety of disease like chronic kidney disease, cancer, and tuberculosis (10, 12, 42, 48). Along this line, ANO9 participates in signaling events downstream of the single immunoglobulin interleukin-1 related receptor (SIGIRR) to antagonize interleukin and toll like receptor mediated signaling, a mechanism to dampen immunological and inflammatory processes (48–50). Defects within the anti-inflammatory/immunosuppressive SIGIRR/ANO9 pathway cause aberrant CD4+ T-cells, leading to inflammatory diseases and anti-donor reactive T-cells in renal transplantation (50–52).

## Methods

### Animals

Isolation of mouse lymphocytes was approved by the local Ethics Committee of the Government of Unterfranken/Wuerzburg (AZ: 55.2-2532-2-1596). Animals were hosted on a 12:12 h light:dark cycle under constant temperature (24 ± 1°C) in standard cages. They were fed a standard diet with free access to tap water. C57BL/6 females in the age around 20 weeks were used for lymphocyte isolation.

### Cell culture, transfection, RT-PCR

Jurkat E6.1 cells (ATCC-TIB-152, ATCC, Manassas, VA, USA) were cultured in RPMI1640 supplemented with 10% FBS, 2 mM L-glutamine, 1% sodium pyruvate solution (100 mM), 1% NEM nonessential amino acids solution (x100), 1% HEPES buffer solution (1M) and 1% penicillin/streptomycin (Capricorn Scientific, Ebsdorfergrund, Germany). Cell transfection was carried out by electroporation using the Neon™ transfection system (Invitrogen, ThermoFisher Scientific, Schwerte, Germany) and siRNAs against ANO6 (TMEM16F), ANO9 (TMEM16J), ATP2B1, ATP2B4, DLG1, PKP1 or SIGIRR (**Table 1**, Stealth siRNA, Invitrogen, Silencer Select, ambion, ThermoFisher Scientific, Schwerte, Germany);. Experiments were performed 48–72 h after transfection. For Ca^2+^ measurements, 800.000 cells (60 µl) were mixed with 120 µl Corning Matrigel matrix (Merck, Sigma-Aldrich, Darmstadt, Germany) and attached to glass coverslips.

**Table 1:** RT-PCR Primer and siRNA.

Gene accession number	Primer	Size (bp)
ATP2B1 NM_001001323.2	s: 5´- CAGGTCCACAGATGCATTACG as: 5´- GTTCTTGTTCAATTCGGCTCTG	455
ATP2B4 NM_001001396.3	s: 5´- CACTATGGAGGTGTACAGAATC as: 5´- GTCACTAACACCACGATGATC	359
IL-2 NM_000586.4	s: 5´- CAGTGCACCTACTTCAAGTTC as: 5´- CTAAATTTAGCACTTCCTCCAG	221
IP3R1 NM_001099952.4	s: 5´- GCGAAAGGAGAAGAGAATAAAG as: 5´- GTTCAAAGTCAAGAGCACCTG	367
IP3R2 NM_002223.4	s: 5´- CCCCCATGTCATCATACTTTG as: 5´- CTCCATTCTCATAGTTGCTGC	726
IP3R3 NM_002224.4	s: 5´- CCCCTGCCTTCGACTCTAC as: 5´- CTGCTTCTTCCTCATTTGCTC	542
ORAI1 NM_032790.4	s: 5´- TGAGCCTCAACGAGCACT as: 5´- GAACTTGACCCAGCAGAGC	407
ORAI2 NM_001126340.3	s: 5´- CGCAGCTACCTGGAACTGG as: 5´- CTCGATCTCGCGGTTGTGG	612
ORAI3 NM_152288.3	s: 5´- CGGCTACCTGGACCTCATG as: 5´- GAGATTGGAAGCTGGACTAAG	532
PDCD1, PD-1 NM_005018.3	s: 5´- GCTTCGTGCTAAACTGGTACC as: 5´- GACAATGGTGGCATACTCCG	572
PDCD1LG1, PDL1, CD274 NM_014143.4	s: 5´- CTGTCTTTATATTCATGACCTAC as: 5´- GTGTTGATTCTCAGTGTGCTG	589
PDCD1LG2, PDL2 NM_025239.4	s: 5´- GAAGTCATGTGAACCTTGGAG as: 5´- GTGAAGCAGCCAAGTTGGATG	533
RYR1 NM_000540.3	s: 5´- GAACCCGACCCTGACTACG as: 5´- CCACACCATGTAGCAGTTGC	510
RYR2 NM_001035.3	s: 5´- GATGCTGATTCTGACTTTGAGG as: 5´- CATTGGGACTTGTAGCTTGTG	643
RYR3 NM_001036.6	s: 5´- CAGAAAAGGAAACAGATGCAAG as: 5´- GGACTGGGTTCTTCTCTTCAC	517
ANO1; TMEM16A, NM_018043	s: 5´- CGACTACGTGTACATTTTCCG as: 5´- GATTCCGATGTCTTTGGCTC	445
ANO2; TMEM16B NM_001278596	s: 5´- GTCTCAAGATGCCAGGTCCC as: 5´- CTGCCTCCTGCTTTGATCTC	553
ANO3; TMEM16C NM_001313726	s: 5´- cttccctcttccagtcaac as: 5´- aaacatgatatcggggcttg	461
ANO4; TMEM16D NM_001286615	s: 5´- CGGAAGATTTACAGGACACCC as: 5´- GATAACAGAGAGAATTCCAATGC	505
ANO5; TMEM16E NM_213599	s: 5´- gaatgggacctggtggac as: 5´- gagtttgtccgagcttttcg	713
ANO6; TMEM16F NM_001025356	s: 5´- GGAGTTTTGGAAGCGACGC as: 5´- GTATTTCTGGATTGGGTCTG	325
ANO7; TMEM16G NM_001370694	s: 5´- CTCGGGAGTGACAACCAGG as: 5´- CAAAGTGGGCACATCTCGAAG	470
ANO8; TMEM16H NM_020959	s: 5´- ggaggaccag ccaatcatc as: 5´- tccatgtcattgagccag	705
ANO9; TMEM16J NM_001012302	s: 5´- GCAGCCAGTTGATGAAATC as: 5´- GCTGCGTAGGTAGGAGTGC	472
ANO10; TMEM16K NM_018075	s: 5´- GTGAAGAGGAAGGTGCAGG as: 5´- GCCACTGCGAAACTGAGAAG	769
GAPDH NM_001289726	s: 5´-GTATTGGGCGCCTGGTCAC as: 5´-CTCCTGGAAGATGGTGATGG	200
siRNA ANO6; TMEM16F, Stealth, HSS176378, Invitrogen	5´- CCUCCAUCAUCAGCUUUAUAAUUAU	
siRNA ANO9; TMEM16J, Stealth; HSS155265-7, Invitrogen	5´-UGAAGUACCAGAGGCUGCGUGAGAA 5´-CCAUGCCUGAAAGAAGGCAACUCUA 5´-GGACUUCCAGGACCCUGAUGGGAUU	
siRNA ATPB1; Silencer Select, s753, ambion	5´-CAGUUAUCAUUCUAGUAUUTT	
siRNA ATPB4; Silencer Select, s1759, ambion	5´-GGACUAUCUGCAUAGCUUATT	
siRNA DLG1 Silencer Select, s4121, ambion	5´-GAUGAUGAAUAGUAGUAUUTT	
Scrambled siRNA, Silencer Select, negative control #1 siRNA, ambion		

### Flow cytometry and proliferation assay

Jurkat cells activated with CD3AB (Monoclonal Antibody, OKT3, eBioscience™, 14-0037-82, Invitrogen or CD3AB and phorbol-12-myristate-13-acetate and phytohemagglutinin (PP; 5 ng/mL/10 µg/mL) and treated with siRNA against ANO9 were collected and washed with cold Dulbecco’s PBS (DPBS), and centrifuged at 500 g and 4°C for 10 min. Cells were incubated for 10 min in 100 µL annexin binding buffer containing 5 µL annexin V-FITC and 2.5 µL 7-aminoactinomycin D (7-AAD; BioLegend, San Diego, CA, USA) with 10 µmol/l ionomycin or DMSO as a control. Reactions were stopped by adding 400 µL of DPBS, and cells were immediately analyzed using a BD Accuri™C6 flow cytometer. The total events collected were at least 20,000 events per sample. Cell proliferation was assessed by cell counting in a Neubauer chamber (daily aliquots) and results were confirmed in assays using 3‐(4,5‐dimethylthiazol‐2‐yl)‐2,5‐diphenyl‐ 2H‐tetrazolium bromide (MTT, M2128, Sigma‐Aldrich, Taufkirchen, Germany).

### Isolation of mouse lymphocytes

Peripheral lymph nodes from C57BL/6J mice (Charles River Laboratories, Sulzfeld, Germany) were harvested and used tp prepare single-cell suspensions using a 100 µm sieve and a centrifugation step (500g, 15 min at 4°C). Primary cells were cultured in RPMI 1640 (Capricorn RPMI-XA), 10%FBS (Capricorn), 1% glutamine (Capricorn STA-B), 50 µM ß-mercaptoethanol (31350010, Gibco), 20 mM HEPES (Capricorn HEP-B), 1mM sodium pyruvate (Capricorn NPY-B), 1% MEM-vitamin (M6895, Sigma), 1% Pen-Strep (Capricorn PS-B) for 24h activated with mCD3 AB (5 µg/ml, hamster anti mouse CD3e, BD Pharmingen Cat: 553058 Lot 3030391, BD Biosciences), and mCD28 AB (5 µg/ml, anti-mouse CD28 purified *in vivo* GOLD, Leinco Technologies, inc Cat C379 Lot 1223L780). For measurements of intracellular calcium concentrations, 100.000 cells were seeded in Matrigel (Corning).

### RT-PCR

For semiquantitative RT-PCR total RNA was isolated using NucleoSpin RNA II columns (Macherey-Nagel). Total RNA (0.5 μg/25 μl reaction) was reverse-transcribed using random primers (Promega) and M-MLV Reverse Transcriptase RNase H Minus (Promega). Each RT-PCR reaction contained sense (0.5 μM) and antisense primers (0.5 μM), 0.5 μl cDNA and GoTaq Polymerase (Promega). After 2 min at 95°C, cDNA was amplified (targets 30 cycles, reference GAPDH 25 cycles) for 30 s at 95°C, 30 s at 56°C and 1 min at 72°C. PCR products were visualized by loading on Midori Green Xtra (Nippon Genetics Europe) containing agarose gels and were analyzed densitometrically by relating the density of the target bands to that of the GAPDH bands using Image J 1.52r software (NIH).

### Western blotting

Protein was isolated from Jurkat cells using RIPA-buffer (#9806, cell signaling) with 1 mM PMSF. After quantification, proteins were separated by 8.5% SDS-PAGE and transferred to a PVDF membrane (GE Healthcare, Munich, Germany). Membranes were incubated overnight at 4°C with primary antibodies against NFATC1 (Monoclonal Antibody (7A6), MA3-024, invitrogen) or Phospho-NFATC1 (monoclonal Mouse IgG, Clone # 679340, MAB5640, R&D systems, Abingdon, UK) in 3% (w/v) NFM/TBST-T). Membranes incubated afterwards with horseradish peroxidase (HRP)-conjugated goat anti-rabbit or sheep anti-mouse secondary antibodies at room temperature for 2 h and immunoreactive signals were visualized using a SuperSignal HRP Chemiluminescence Substrate detection kit (#34577; Thermo Fisher Scientific, Waltham, Massachusetts, USA).

### IL-2 release

Cellular release of IL-2 was detected using quantikine colorimetric sandwich ELISA kit (D2050, R&D Systems, Wiesbaden-Nordenstadt, Germany). Transfected jurkat E6.1 cells were treated with PHA/PMA for 24h. Supernatants were collected, centrifugated and IL-2 release was measured according to the manufacturing protocol. The signals were detected using the microplate reader NOVOstar (BMG Labtech, Offenburg, Germany).

### Immunofluorescence

Jurkat E6.1 cells were fixed with 4% paraformaldehyde, centrifuged, washed with PBS, permeabilized and blocked with 0.04% Triton X-100 and 5% BSA for 1h at 37°C. Cells were centrifuged, washed with PBS and incubated with primary antibodies against ANO9 (Peptide sequence: RPPMPAHPTPASIFSARSTD, Davids Biotechnologie, Regensburg, Germany), ORAI1 (anti-Orai1 antibody, mouse monoclonal, SAB4200273, Sigma-Aldrich), SERCA (goat anti SERCA3 (N-19), sc-8097, Santa Cruz) or PMCA (anti-PMCA, mouse monoclonal, clone 5F10, MA3-914, Invitrogen) in 0.5% BSA for 1h at 37°C and subsequent after centrifugation with a secondary antibodiese donkey anti mouse Alexa Fluor 488, donkey anti goat Alexa Fluor 488 or goat anti-rabbit Alexa 660 (A21202, A11055, A21074, Invitrogen, ThermoFisher Scientific, Schwerte, Germany) for 1h at 37°C. Cells were counterstained with Hoe33342 (Sigma-Aldrich, Merck, Darmstadt, Germany). Immunofluorescence was detected using an Axiovert Observer microscope equipped with ApoTome2 and ZEN 2.6 (blue edition) Software (Carl Zeiss Microscopy Deutschland GmbH, Oberkochen, Germany). Colocalization analysis was done using ZEN Microscopy Software. Briefly, every pixel in the image is plotted in the displayed scatter diagram based on its intensity level from each channel. The software estimates the degree of colocalization according to specialized algorithms within the selected region of interest (ROI). The ROI was set to the plasma membrane. Colocalization was estimated by Pearson’s correlation coefficient. Its values range between +1.0 and -1.0, where 0 indicates no significant correlation, while +1.0 and −1.0 indicate 100% positive and negative correlation, respectively.

### Measurement of intracellular Ca^2+^ [Ca^2+^]_i_

Jurkat E6.1 cells in Matrigel matrix on glass cover slips were loaded with 2 µM Fura-2/AM (Biozol, Eching, Germany) and 0.02% Pluronic F-127 (Invitrogen, Darmstadt, Germany) in ringer solution (mmol/l: NaCl 145; KH_2_PO_4_ 0,4; K_2_HPO_4_ 1,6; Glucose 5; MgCl_2_ 1; Ca^2+^-Gluconat 1,3) for 1h at room temperature. Fluorescence was detected in cells perfused with Ringer’s solution at 37°C using an inverted microscope (Axiovert S100, Zeiss, Germany) and a high speed polychromator system (VisiChrome, Puchheim, Germany). Fura-2 was excited at 340/380 nm, and emission was recorded between 470 and 550 nm using a CoolSnap camera (CoolSnap HQ, Visitron). [Ca^2+^]*_i_* was calculated from the 340/380 nm fluorescence ratio after background subtraction. The formula used to calculate [Ca^2+^]*_i_* was [Ca^2+^]*_i_ =Kd* x (*R*-*R*_min_)/(*R*_max_-*R*) x (S_f2_/S_b2_), where *R* is the observed fluorescence ratio. The values *R*_max_ and *R*_min_ (maximum and minimum ratios) and the constant S_f2_/S_b2_ (fluorescence of free and Ca^2+^-bound Fura-2 at 380 nm) were calculated using 2 µmol/liter ionomycin (Cayman Chemical, Biomol GmbH, Hamburg, Germany) and 5 mmol/liter EGTA to equilibrate intracellular and extracellular Ca^2+^ in intact Fura-2-loaded cells. The dissociation constant for the Fura-2•Ca^2+^ complex was taken as 224 nmol/liter. ER Ca^2+^ signals were detected in Ca^2+^ sensor ER-LAR-GECO1 expressing jurkat cells. Jurkat cells were excited at 560 nm and emission was recorded between 620 ± 30 nm. Control of the experiment, imaging acquisition, and data analysis were done with the software package Meta-Fluor (Universal 26 imaging, USA). For every series of experiments, Ca^2+^ signals from about 100 cells fixed on 5-15 different glass cover slips were measured.

### Patch clamping

Jurkat cells were fixed on poly-L-lysine coated cover slips. Coverslips were mounted on an inverted microscope (Axiovert 100, Zeiss, Oberkochen). Patch pipettes were filled with a cytosolic-like solution containing (in mM) KCl 30, K-gluconate 95, NaH2PO4 1.2, Na2HPO4 4.8, EGTA 1, Ca -gluconate 0.758, MgCl2 1.03, D-glucose 5, ATP 3, pH 7.2. The intracellular (pipette) Ca2+ activity was 0.1 µM. The bath was perfused continuously with Ringer solution at a rate of 8 ml/min. Patch pipettes had an input resistance of 4–6 MΩ and whole cell currents were corrected for serial resistance. Currents were recorded using a patch clamp amplifier EPC9, and PULSE software (HEKA, Lambrecht, Germany) as well as Chart software (AD Instruments, Spechbach, Germany). The currents were corrected for the serial resistance. The acquisition frequency was 1 kHz. The signal was filtered by a low-pass Bessel filter. In regular intervals, the membrane voltage (Vc) was clamped from -100 to +100 mV in steps of 20 mV. If not voltage clamped, the cells were kept at their intrinsic membrane voltage in the current clamp mode. Membrane capacitance was measured using the PULSE software. Current density was calculated by dividing whole cell currents by cell capacitance.

### Materials and statistical analysis

Chemicals were purchased from Sigma-Aldrich or Calbiochem (Merck, Darmstadt, Germany). Statistical analysis was performed using Student’s t-test (for paired or unpaired samples as appropriate) or ANOVA with Benferroni-Holm posthoc test. For the assessment of the intracellular [Ca^2+^], typically 50 – 100 cells were measured per cover slip and the mean value for these measurements was counted as one experiment. A value of p<0.05 was accepted as a significant difference. Data are reported as means ± SEM.

## Data Availability

The raw data supporting the conclusions of this article will be made available by the authors, without undue reservation.
