# Supplementary material for: Anoctamin 9 determines Ca2+ signals during activation of T-lymphocytes
Source: Front Immunol. 2025 Mar 26;16:1562871. doi: 10.3389/fimmu.2025.1562871 (PMC11979140; doi:10.3389/fimmu.2025.1562871)
Supplement: Supplementary file 1 [file DataSheet1.pdf]

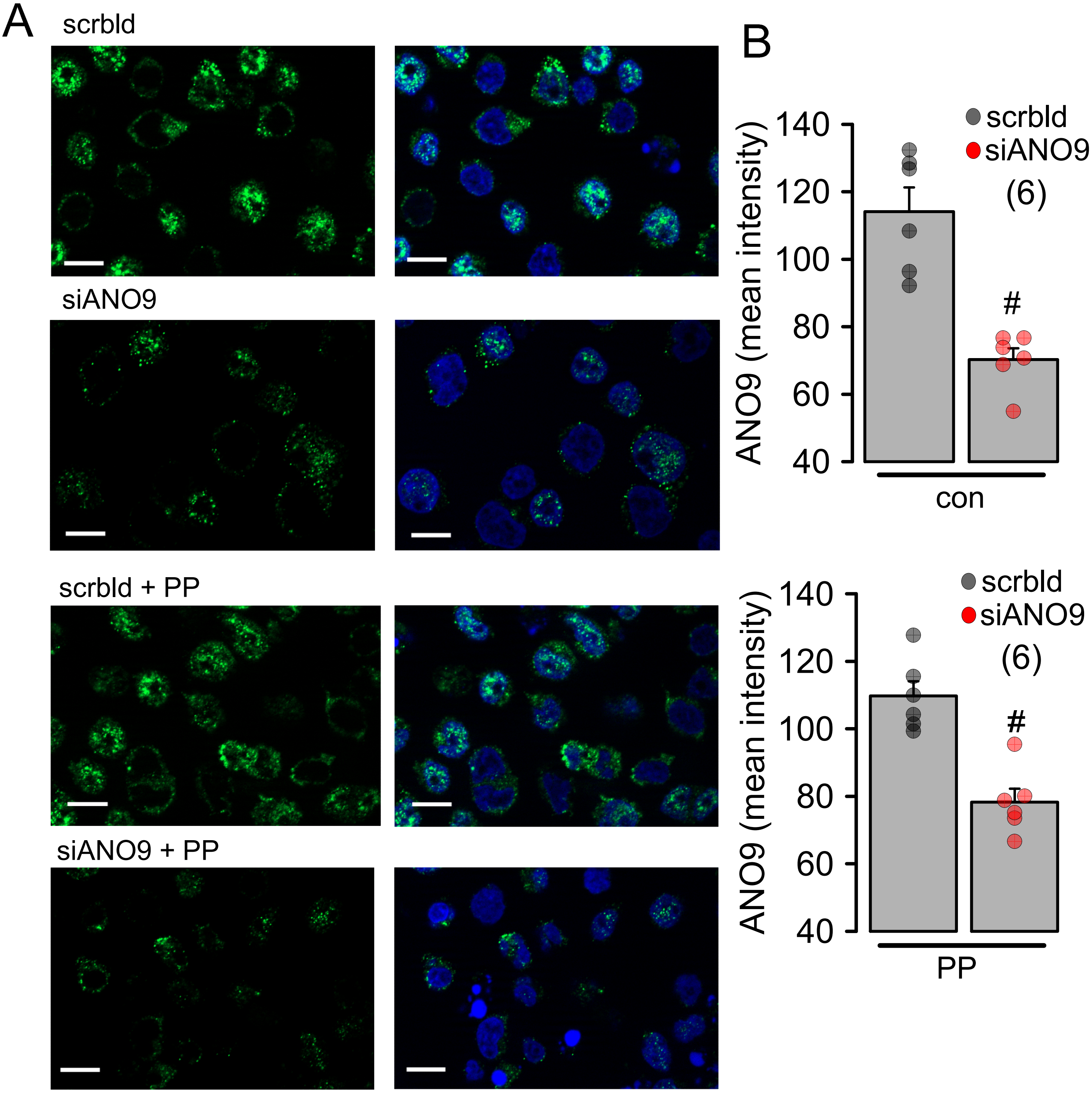

Supplementary Figure 1. *Immunolabeling of ANO9 in Jurkat T-cells indicates knockdown of ANO9-expression by siRNA.* **A)** ANO9 immunofluorescence labeling using a rabbit anti-human ANO9 antibody (Invitrogen, PA5-69330) and a secondary fluorescent anti-rabbit antibody. **B**) Analysis of immunofluorescence intensity in control cells and after stimulation of the cells with PMA (50 ng/ml) and PHA (PP; 1 µg/ml). Mean ± SEM (number of slides, each slide represents the mean of 20 cells measured). #significant difference compared to scrambled (scrbld) ($p < 0.05$; unpaired t-test).
