# Supplementary material for: Anoctamin 9 determines Ca2+ signals during activation of T-lymphocytes
Source: Front Immunol. 2025 Mar 26;16:1562871. doi: 10.3389/fimmu.2025.1562871 (PMC11979140; doi:10.3389/fimmu.2025.1562871)
Supplement: Supplementary file 2 [file DataSheet2.pdf]

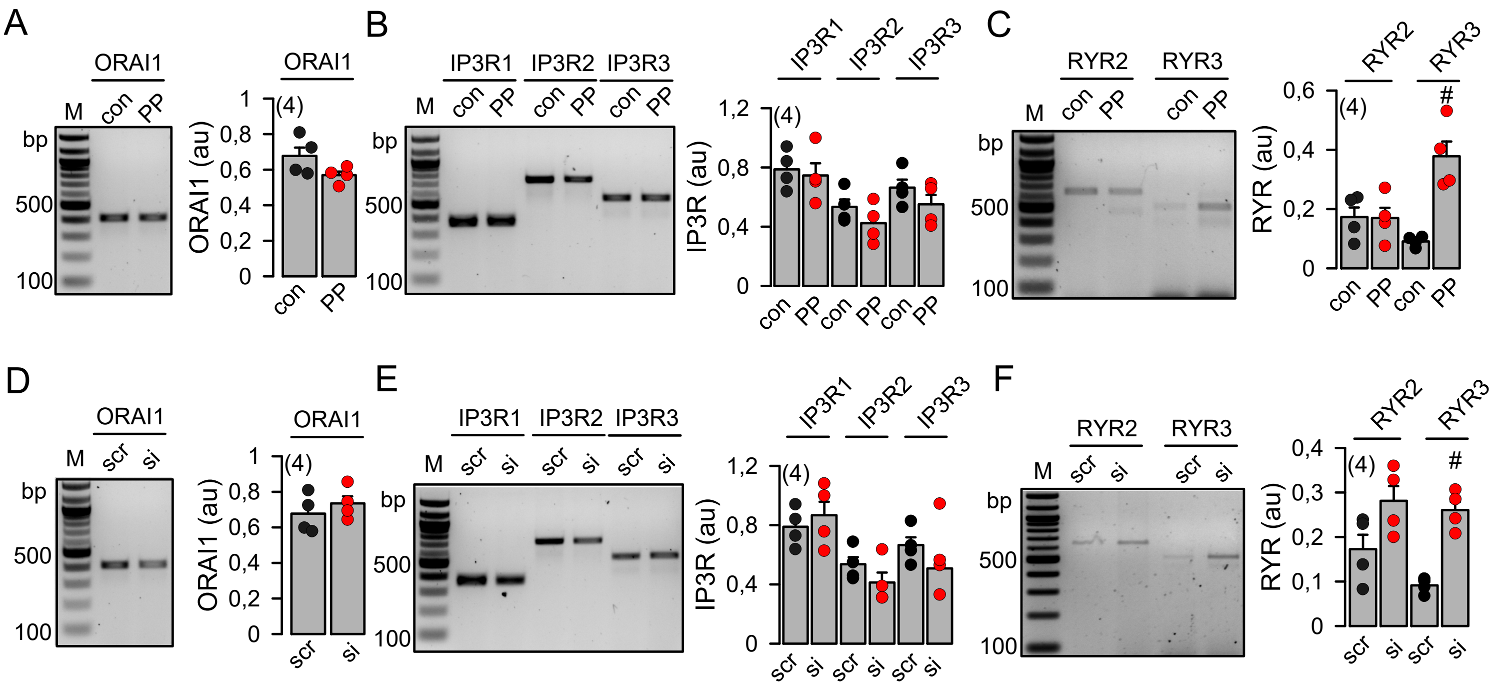

Supplementary Figure 2. *Activation of Jurkat T-cells by PP and knockdown of ANO9 expression have minor effects on expression of* Ca^{2+} *regulating proteins.* **A-C)** Semiquantitative RT-PCR analysis of the effects of PP-activation of Jurkat T-cells on expression of ORAI1, IP_3 receptors 1-3 (IP_3R1-3) and ryanodine receptor 2 and 3 (RYR2,3). **D-F)** Semiquantitative RT-PCR analysis of the effects of siRNA-ANO9 (si) on expression of ORAI1, IP_3 receptors 1-3 (IP_3R1-3) and ryanodine receptor 2 and 3 (RYR2,3). Mean ± SEM (number of experiments). #significant difference when compared to control (con) or scrambled (scr) ($p < 0.05$; unpaired t-test).
