# Supplementary material for: Anoctamin 9 determines Ca2+ signals during activation of T-lymphocytes
Source: Front Immunol. 2025 Mar 26;16:1562871. doi: 10.3389/fimmu.2025.1562871 (PMC11979140; doi:10.3389/fimmu.2025.1562871)
Supplement: Supplementary file 3 [file DataSheet3.pdf]

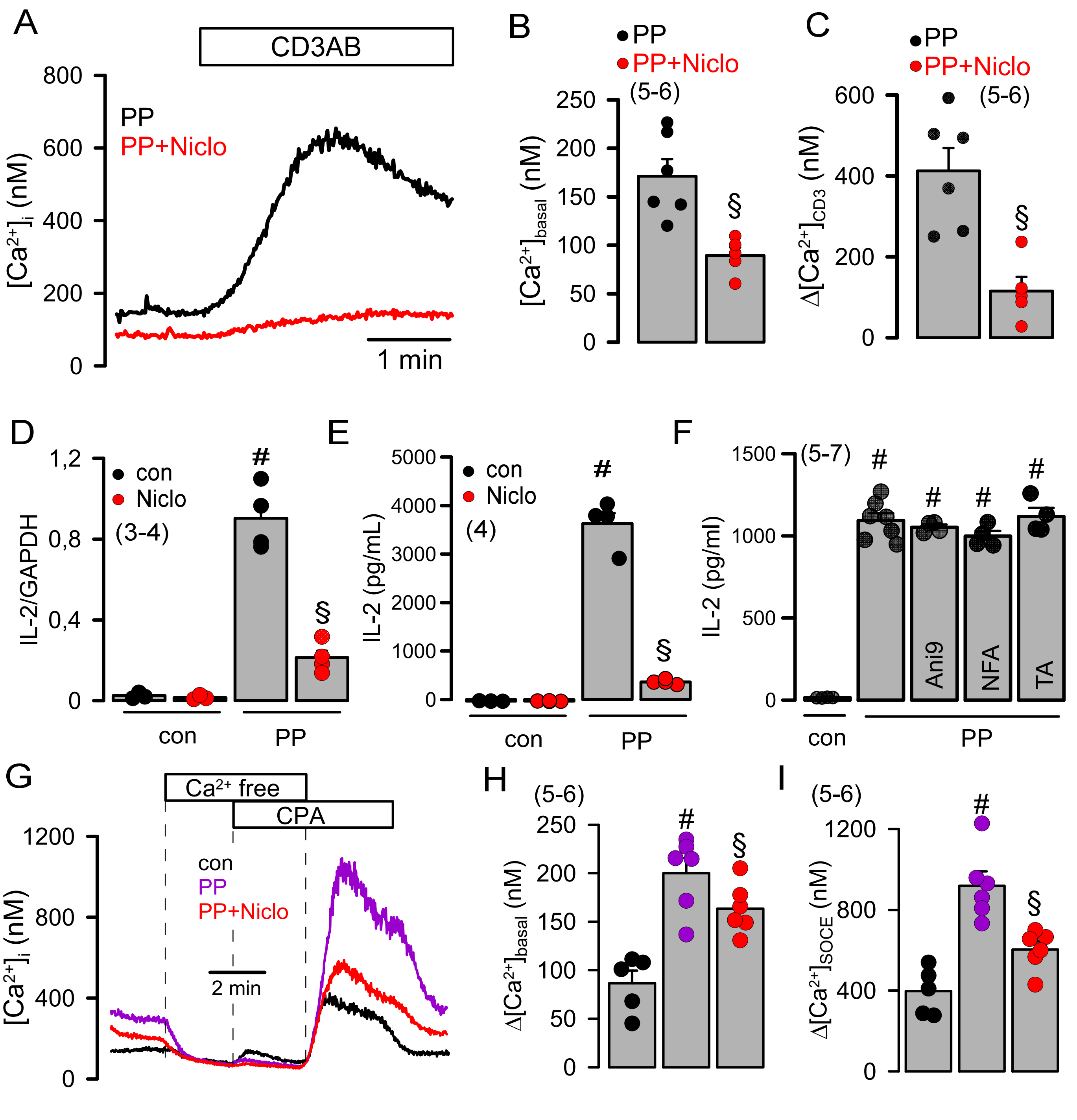

Supplementary Figure 3. *Inhibition of Ca^{2+} influx and IL-2 release by niclosamide.* **A-C**) Activation of Ca^{2+} influx by CD3-AB (2.5 µg/ml) and inhibition by niclosamide (Niclo; 500 nM). (number of measurements/cell batches). **D,E**) Inhibition of IL-2 transcription and IL-2 release by niclosamide in Jurkat cells activated by PMA 50 ng/ml) and PHA (1 µg/ml) (PP). (number of assays). **F**) Lack of inhibition of IL-2 release by Ani9 (1 µM), niflumic acid (NFA; 5 µM), and tannic acid (TA; 5 µM) in cells activated by PP (number of assays). **G**) $[Ca^{2+}]_i$ in Jurkat cells under control conditions and after PP-activation in the absence or presence of Niclo. ER store emptying by CPA (10 µM) in extracellular Ca^{2+} free buffer. **H,I**) Summary of the change in basal $[Ca^{2+}]_i$ and SOCE. (number of measurements). Mean ± SEM, #significant activation by PP ($p < 0.05$; ANOVA). §significant inhibition by Niclo ($p < 0.05$; unpaired t-test and ANOVA).
