# Supplementary material for: Anoctamin 9 determines Ca2+ signals during activation of T-lymphocytes
Source: Front Immunol. 2025 Mar 26;16:1562871. doi: 10.3389/fimmu.2025.1562871 (PMC11979140; doi:10.3389/fimmu.2025.1562871)
Supplement: Supplementary file 4 [file DataSheet4.pdf]

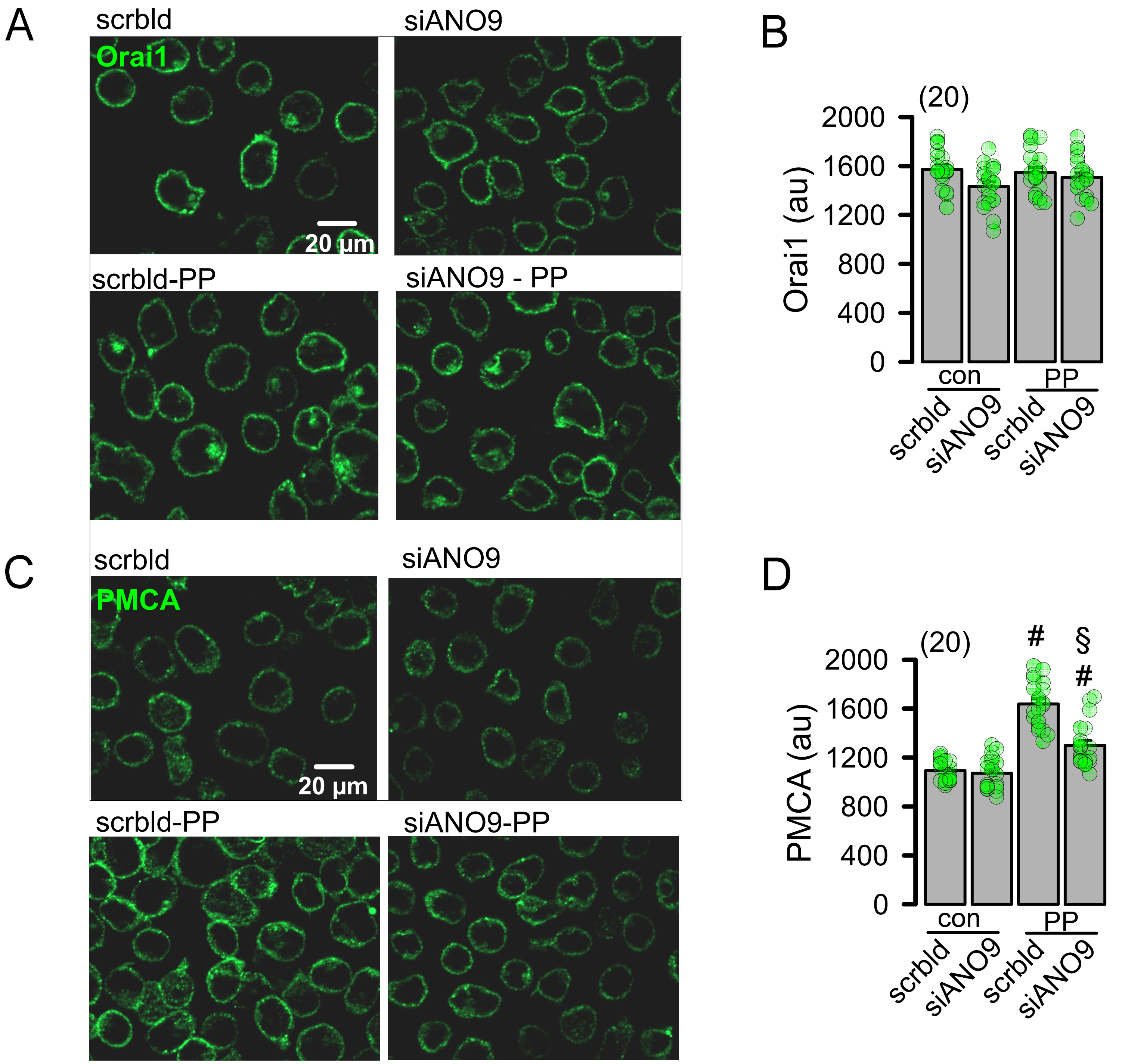

Supplementary Figure 4. *Membrane expression of PMCA but not Orai1 depends on expression of ANO9.* **A**) Staining of Orai1 in Jurkat T-cells in the absence or presence of PP (5 ng/mL/10 µg/mL) and after treatment with scrambled (scrbld) RNA or siRNA-ANO9. **B**) Summary of fluorescence intensities (arbitrary units, au). **C**) Staining of PMCA in Jurkat T-cells in the absence or presence of PP (5 ng/mL/10 µg/mL) and after treatment with scrambled (scrbld) RNA or siRNA-ANO9. **D**) Summary of fluorescence intensities (arbitrary units, au). Mean ± SEM (number of slides, each slide represents the mean of 20 cells measured). #significant increase by PP ($p<0.05$; unpaired t-test). §significant difference compared to scrbld ($p<0.05$; unpaired t-test).
