# Supplementary material for: Anoctamin 9 determines Ca2+ signals during activation of T-lymphocytes
Source: Front Immunol. 2025 Mar 26;16:1562871. doi: 10.3389/fimmu.2025.1562871 (PMC11979140; doi:10.3389/fimmu.2025.1562871)
Supplement: Supplementary file 6 [file DataSheet6.pdf]

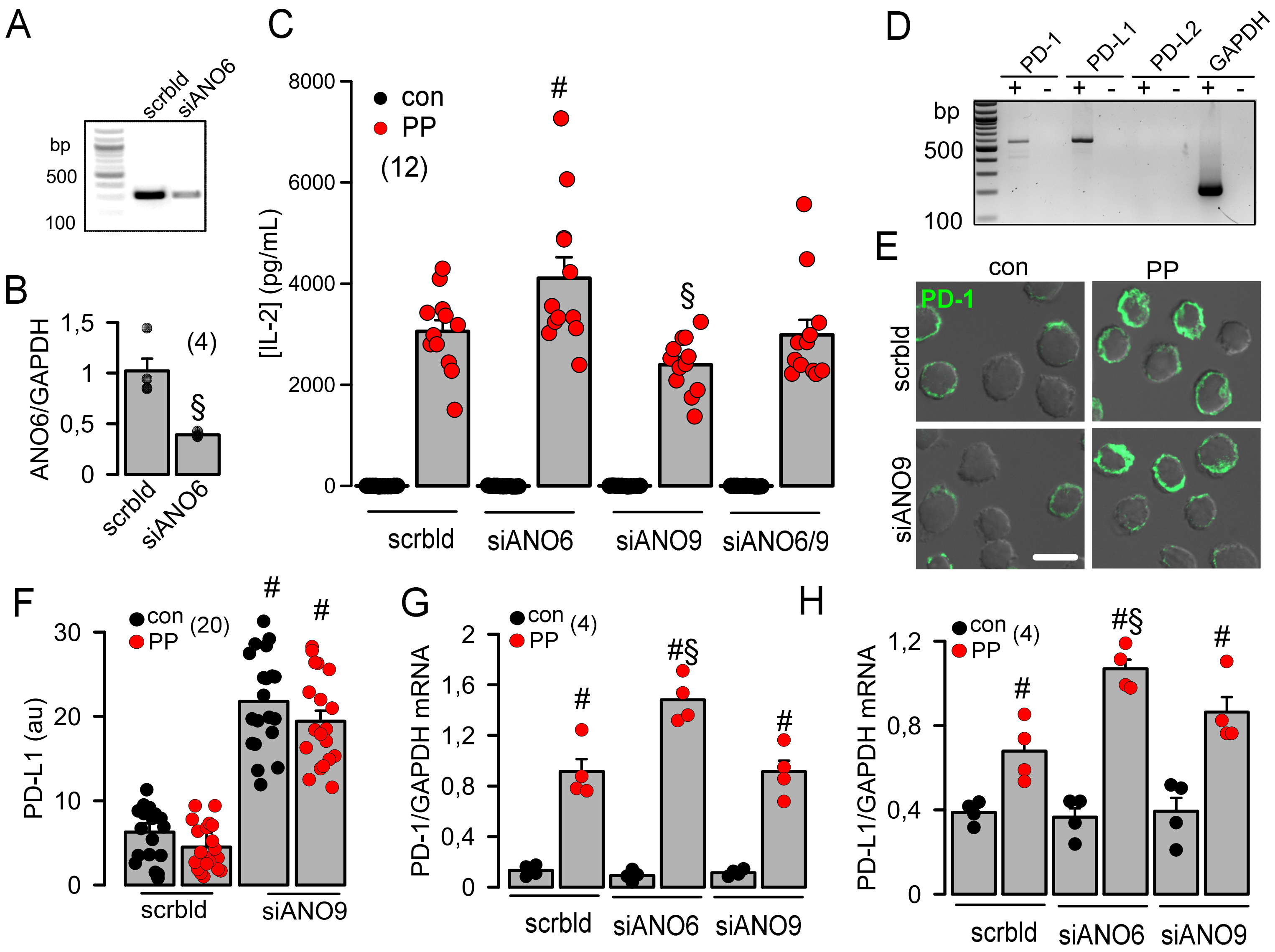


**Supplementary Figure 6**. *The effect of ANO9 on activation of T-cells is independent of ANO6 and programmed cell death 1.* **A,B**) RT-PCR analysis of ANO6-expression and knockdown by siRNA. **C**) Effects of PP-activation and siRNA-knockdown of ANO6 and ANO9 on IL-2 release (ELISA). **D**) RT-PCR analysis demonstrating expression of PD-1 and PD-L1/L2 in the absence of PP. **E**) PD-1 staining in Jurkat cells under control conditions and after activation by PP in the absence of presence of siRNA-ANO9. **F**) Summary of green PD-1 fluorescence (au. arbitrary units. **G,H**) RT-PCR analysis of the effects of PP and siRNA-knockdown of ANO6 and ANO9 on the expression of PD1 and PD-L1. Mean ± SEM (number of experiments). #significant increase by PP ($p<0.05$; unpaired t-test). #significant change by siRNA-knockdown ($p<0.05$; unpaired t-test).
