# Supplementary material for: Anoctamin 9 determines Ca2+ signals during activation of T-lymphocytes
Source: Front Immunol. 2025 Mar 26;16:1562871. doi: 10.3389/fimmu.2025.1562871 (PMC11979140; doi:10.3389/fimmu.2025.1562871)
Supplement: Supplementary file 7 [file DataSheet7.pdf]

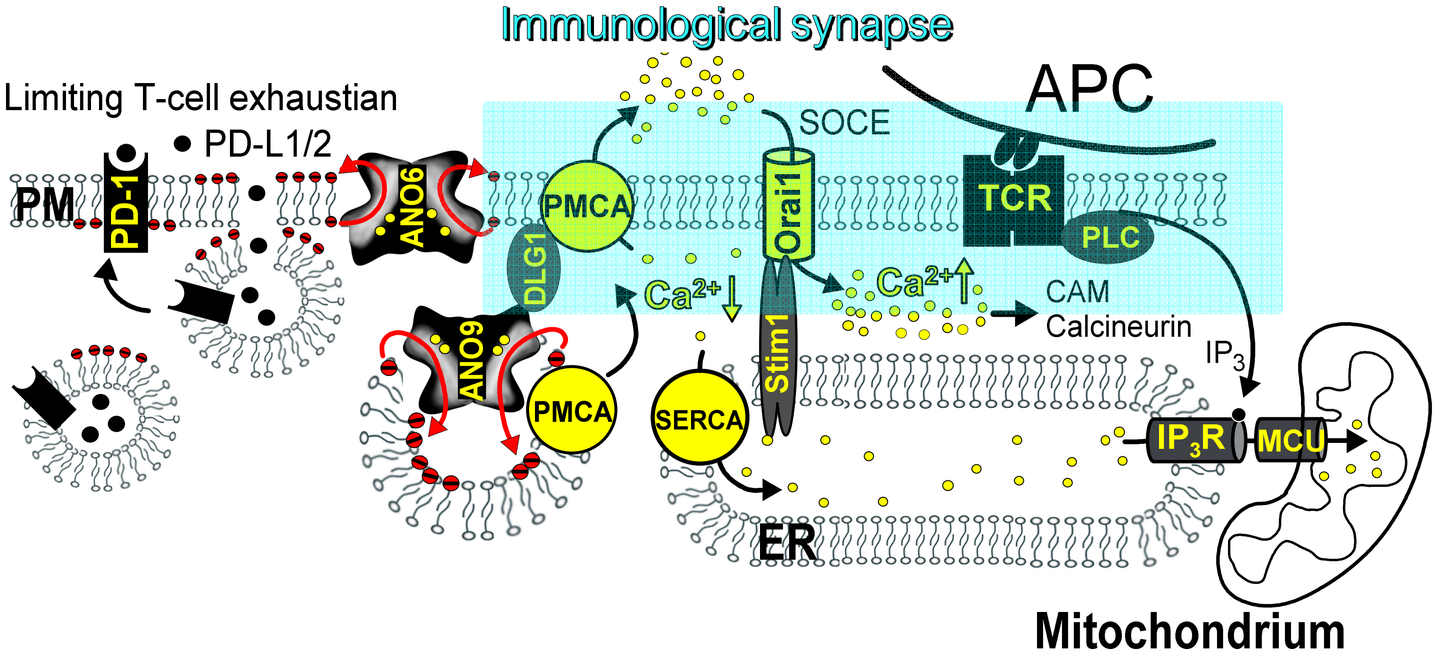

Supplementary Figure 7. *Proposed role of ANO9 and ANO6 in T-cell activation and Ca^{2+} handling.* Activation of T-cells is triggered by binding of antigen presenting cells (APC) to the T-cell receptor (TCR) which generates inositol 1,4,5-trisphosphate (IP_3) through phospholipase C (PLC) and releases Ca^{2+} from the endoplasmic reticulum (ER) Ca^{2+} store. Low ER-Ca^{2+} is sensed by stromal interaction molecule 1 (Stim1), which activates the Ca^{2+} influx channel Orai1. Store-operated Ca^{2+} entry (SOCE) by Orai1 activates CAM/calcineurin-dependent downstream signaling pathways. Due to its scramblase activity and with support by the scaffolding protein discs large 1 (DLG1), ANO9 allows targeting of vesicles transporting the plasma membrane Ca^{2+}-ATP-ase (PMCA) to the immunological synapse. PMCA removes cytosolic Ca^{2+} in close proximity to Orai1 and thus avoids inhibition by high Ca^{2+}. SOCE is also maintained by keeping cytosolic and ER Ca^{2+} low through uptake of Ca^{2+} into the ER via the Ca^{2+} pump SERCA and mitochondrial Ca^{2+} channeling facilitated by IP_3R and the mitochondrial Ca^{2+} uniporter (MCU). In contrast, ANO6 allows exocytosis of programmed cell death 1 (PD-1) receptors and possibly its ligands to terminate T-cell receptor signaling thereby avoiding T-cell exhaustion.
